# Supplementary material for: Transcriptome Analysis Reveals Vimentin-Induced Disruption of Cell–Cell Associations Augments Breast Cancer Cell Migration
Source: Cells. 2022 Dec 13;11(24):4035. doi: 10.3390/cells11244035 (PMC9776984; doi:10.3390/cells11244035)
Supplement: Supplementary file 1 [file cells-11-04035-s001.zip › DEGs and enrichment analysis.zip!/DEGs and enrichment analysis/WT_VIMvsVector KEGG/UP/WT_VIMvsVector_UP.pathway_map.zip!/WT_VIMvsVector_UP.pathway_map/src/hsa04360.html]

hsa04360


- hsa:2042

- Up regulated genes

ENSG00000044524(1.92500348312363)

- hsa:9423

- Up regulated genes

ENSG00000065320(2.57025751012491)

- hsa:10501

- Up regulated genes

ENSG00000167680(2.67271072980687)

- hsa:6387

- Up regulated genes

ENSG00000107562(4.08892060137154)

- hsa:56896

- Up regulated genes

ENSG00000157851(2.5757431187976)

Close
